# S100A7 induction is repressed by YAP via the Hippo pathway in A431 cells

**DOI:** 10.18632/oncotarget.9477

**Published:** 2016-05-19

**Authors:** Yunguang Li, Fei Kong, Junhao Wang, Enze Hu, Rui Wang, Jin Liu, Qianqian Xiao, Weiqing Zhang, Dacheng He, Xueyuan Xiao

**Affiliations:** ^1^ Key Laboratory of Cell Proliferation and Regulation Biology, Ministry of Education, Beijing Normal University, Beijing, China

**Keywords:** S100A7, YAP, Hippo pathway, F-actin, A431

## Abstract

YAP is an oncogenic transcriptional co-activator and is inhibited by the Hippo pathway. Recent studies have revealed that YAP is also a sensor of cell morphology and cell density and can be phosphorylated by cytoskeleton reorganization. Our previous study demonstrated that S100A7 was upregulated in several squamous cell carcinoma (SCC) specimens and was dramatically induced in SCC cells by suspension and dense culture as well as in xenografts. However, little is known about how S100A7 induction occurs in cancer cells. Here, we identify that S100A7 induction is accompanied by YAP phosphorylation in both suspended and dense A431 cells. This correlation reverses after recovery of cell attachment or relief from dense culture. Further examination finds that S100A7 induction is repressed by nuclear YAP, which is further validated by activation or inhibition of the Hippo pathway via loss- and/or gain-of- LATS1 and MST1 function. Strikingly, disruption of the F-actin promotes S100A7 expression via YAP by activation of the Hippo pathway. Furthermore, we demonstrate that repression of S100A7 by YAP required TEAD1 transcriptional factor. Taken together, our findings demonstrate for the first time that S100A7 is repressed by YAP via the Hippo pathway.

## INTRODUCTION

Cutaneous squamous cell carcinoma (SCC) is the second most frequent skin cancer, arises from interfollicular epidermal keratinocytes [1]. S100A7 (psoriasin) was initially identified as a protein that is highly expressed in the skin of patients suffering from psoriasis [2, 3]. In addition, S100A7 is also strongly expressed in differentiated squamous cell carcinoma and skin carcinoma *in situ* as well as keratoacanthoma, whereas it is absent in undifferentiated skin basalioma [4]. Subsequent studies have shown that upregulation of S100A7 is observed in nearly all types of SCC tissues and adenocarcinomas of the breast [4–11]. Recently, we identified that S100A7-negative and -positive cells bi-directionally converted to each other, depending on the cell density and cell morphology in several SCC cells [12, 13]. Importantly, S100A7 was also induced *in vivo* in SCC cells and the expression pattern of S100A7-positive cells in xenografts tissues was similar to that of SCC specimen tissues. However, the mechanisms underlying S100A7 induction both *in vitro* and *in vivo* remains limited, particularly in SCC cells.

The Hippo pathway is a newly established tumor suppressor pathway that limits organ size under physiological conditions [14]. At the core of the Hippo pathway is a kinase cascade consisting of LATS1/2, and MST1/2. MST1/2 kinase phosphorylates and activates the LATS1/2 kinase, the later directly phosphorylates YAP [15–18]. Phosphorylation of YAP (S127) confine it to the cytoplasm, where it can no longer function in target gene expression. Conversely, nuclear YAP is known as a transcriptional coactivator and promotes or represses YAP-dependent gene expression via binding with TEAD. In skin, YAP functions in balancing growth and differentiation during epidermal development [19]. Recently, the Hippo pathway has been recognized to be regulated by cell morphology and cell density via actin cytoskeleton reorganization [20, 21]. Thus, YAP is not simply a growth regulator, but is also a sensor and mediator of cell morphology and cell density.

Many studies to data have focused on identifying genes upregulated by YAP/TAZ [22]. Here, we unequivocally demonstrate that YAP is a repressor of S100A7 induction via the Hippo pathway in A431 cells. Thus, our findings provide new insight for understanding the functions of the Hippo signaling pathway and the actin cytoskeleton in A431 cells.

## RESULTS

### S100A7 induction is accompanied by YAP inactivation, and both are regulated by the cell morphology and cell density in A431 cells

**Figure 1:**
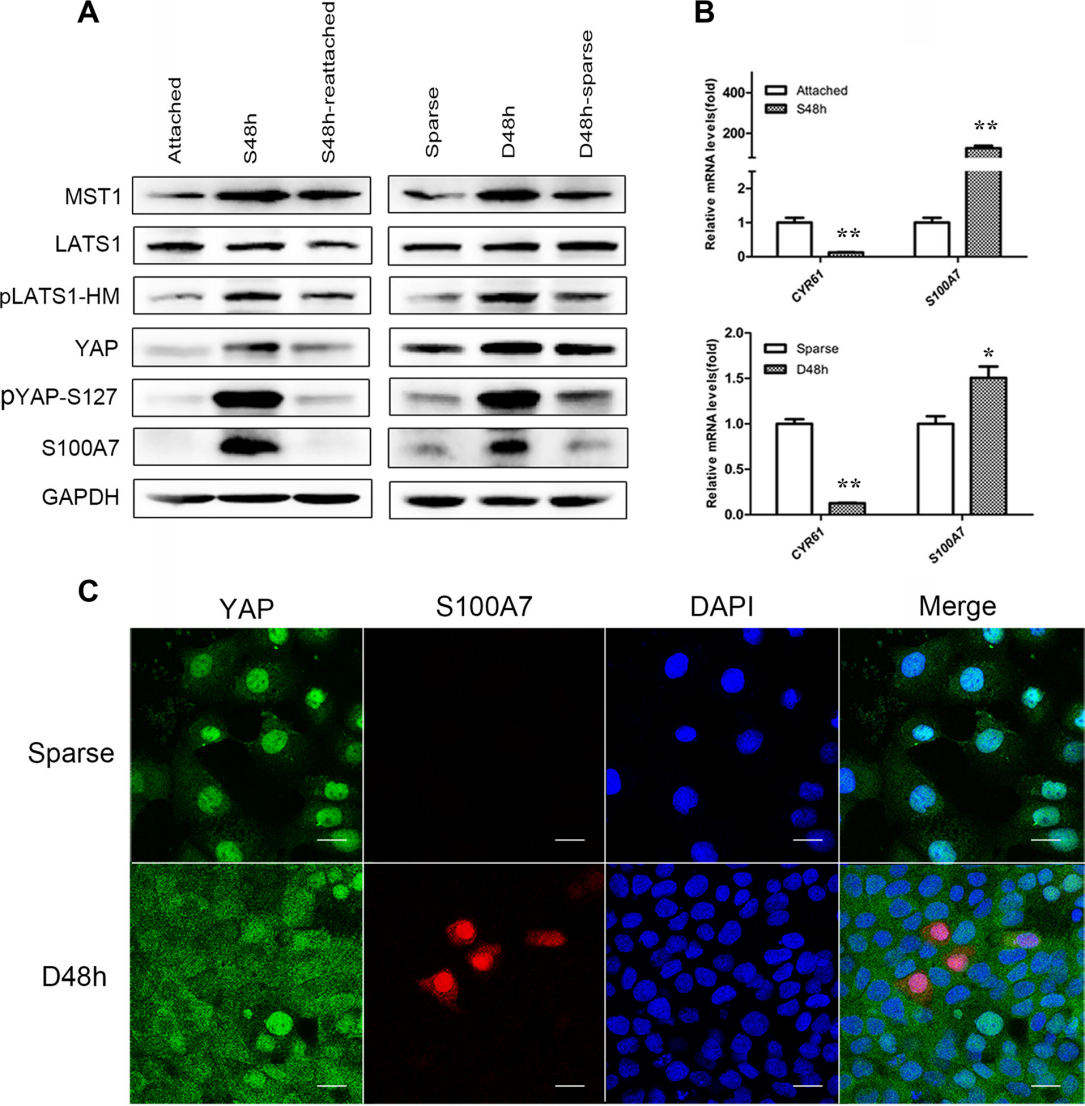
Cell morphology and density regulate S100A7 induction and YAP activity, and subcellular location of S100A7 and YAP are detected in dense cells (**A**) Western blot analyses of S100A7, YAP and pYAP (S127) in the indicated cells. Cells were cultured in suspension for two days (S48 h) and then reattachment for one day (S48 h-reattached). Cells were cultured densely for two days (D48 h) and then relief from dense culture (D48 h-sparse). GAPDH was used as a loading control. (**B**) The expression of *S100A7* and *CTGF* or *CYR61,* two endogenous markers of YAP, were analyzed by qPCR. Error bar, SD of three different experiments.*p < 0.05, **p < 0.01; *t*-test. (**C**) Dense culture induced S100A7 expression and caused YAP nuclear exclusion in A431 cells. Cells were cultured in dense for two days. Samples were then stained with anti-S100A7 (Abcam) and anti-YAP (CST) antibodies. DAPI is a nuclear counterstain. Scale bar, 20 μm.

Our previous studies demonstrated that S100A7 was heterogeneously expressed in A431 cells by cell suspension and confluence culture [12]. However, the mechanism of S100A7 induction is unknown. To gain insight into how S100A7 is induced in A431 cells, we first determined if YAP is involved in S100A7 regulation. To achieve this, A431 cells were cultured in suspension or at two different cell densities, including sparse and dense (Supplementary Figure S1). We compared the expression of S100A7 and YAP in the different culture conditions. As a result, we found that S100A7 induction was accompanied by an increase in the YAP Serine 127 (YAP-S127) phosphorylation in suspended cells compared with attached cells (Figure 1A). Similar phenomena also occurred in dense cells compared with sparse cells (Figure 1A). As shown in Figure 1A, suspension- and dense-mediated S100A7 expression and YAP phosphorylation were dramatically attenuated after recovery of cell attachment or relief from dense culture. We also observed an increase in LATS1 phosphorylation in suspended and dense cells, which indicate that S100A7 may be inhibited by YAP via the Hippo pathway. Consistent with these findings, the level of S100A7 mRNA was significantly increased in suspended and dense cells. In addition, the expression of *CYR61*, a direct endogenous marker of YAP, was analyzed by qPCR as readout of YAP activity. We observed that an increase in YAP phosphorylation resulted in the inhibition of *CYR61* expressions in suspended and dense A431 cells (Figure 1B). These results suggest that nuclear YAP is decreased in suspended and dense A431 cells. Collectively, our data convincingly demonstrate that the dynamic expression of S100A7 is inversely correlated with nuclear YAP in A431 cells. Next, using immunofluorescence, we further examined the expression pattern of S100A7 and YAP. In line with these finding, the percentage of S100A7-positive cells was significantly increased and displayed heterogeneity, whereas YAP markedly translocated to the cytoplasm in dense cells compared with sparse cells, respectively. Representative immunofluorescence images are shown in Figure 1C.

### S100A7 induction is regulated by Hippo-YAP pathway in A431 cells

**Figure 2:**
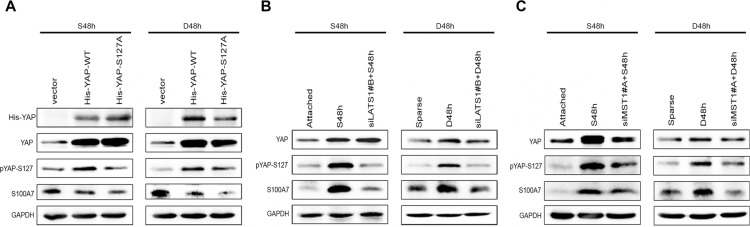
The Hippo pathway is responsible for S100A7 induction in A431 cells (**A**) A431 cells were transfected with YAP-WT and mutant activated YAP-S127A. Subsequently, the cells were cultured in suspension or dense for two days. The expression of S100A7, YAP and pYAP (S127) were detected by Western blotting. GAPDH was used as a loading control. (**B**) siLats1 + S48 h (or D48 h) indicated that cells were cultured in suspension (or dense) for 48 h after silencing of LATS1. C. siMST1 + S48 h (or D48 h) indicated that cells were cultured in suspension (or dense) for 48 h after silencing of MST1.

**Figure 3:**
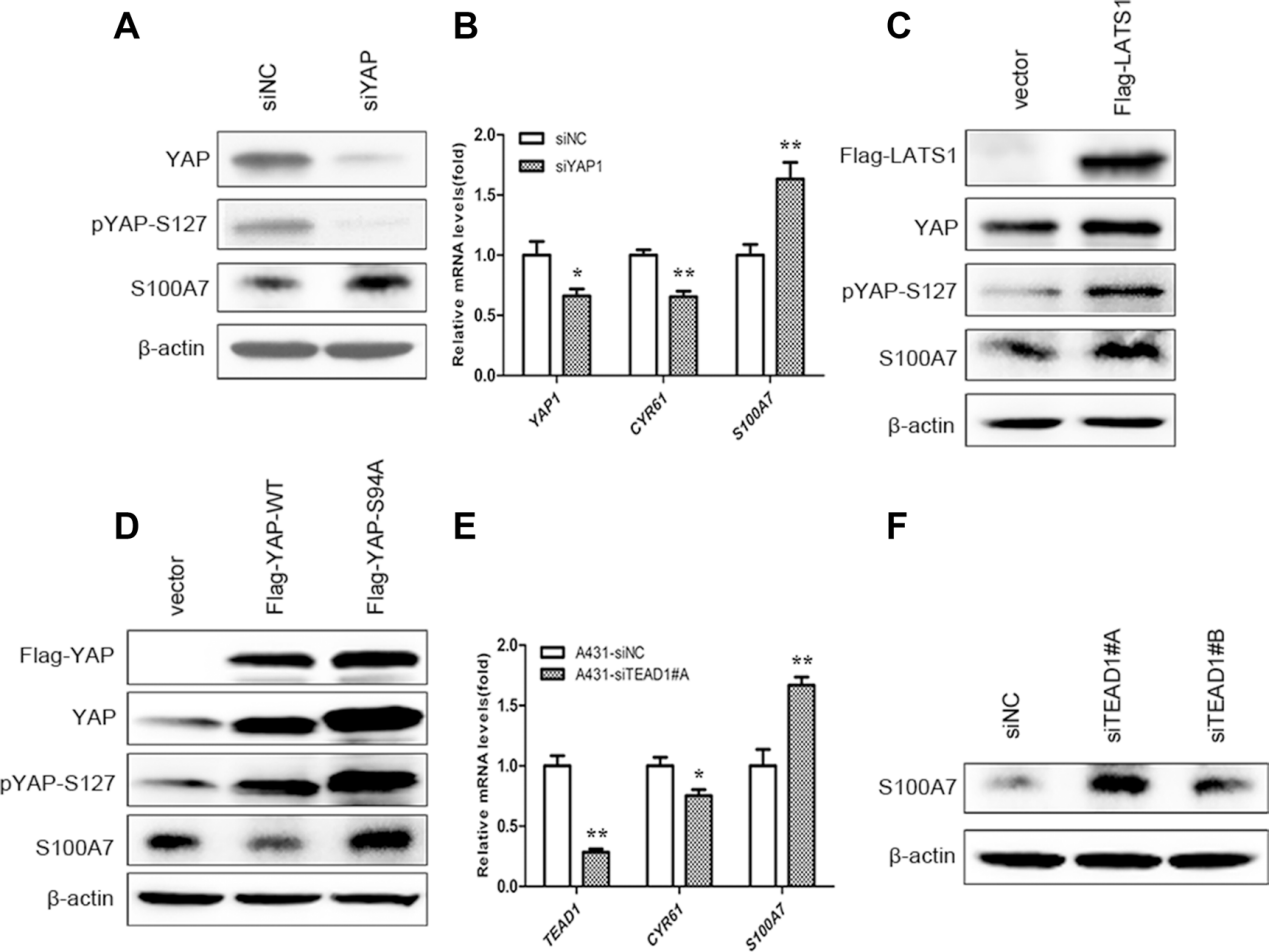
S100A7 induction is regulated by activation of the Hippo pathway in A431 cells, and TEAD1 mediates YAP-dependent S100A7 expression (**A**) Depletion of YAP using siRNA in normal attached A431 cells. The expression of YAP, S100A7 and pYAP (S127) were determined by Western blotting. β-actin was used as a loading control. (**B**) qPCR analyses of *S100A7*, *YAP*, *CTGF* or *CYR61* in the indicated cells after silencing of YAP. Error bar, SD of three different experiments. *p < 0.05, **p < 0.01; *t*-test. (**C**) Overexpression of LATS1 in normal attached A431, cells. Anti-Flag tag antibody was used to judge the transfection efficiency. (**D**) Overexpression of YAP-WT and mutant activated YAP-S94A in normal attached A431 cells. Anti-Flag tag antibody was used to judge the transfection efficiency. (**E**) The mRNA expression of *CYR61*, and *S100A7* were examined using qPCR. Error bar, SD of three different experiments. *p < 0.05, **p < 0.01; *t*-test. (**F**) Western blot analyses for S100A7 expression after TEAD1 silencing in normal attached A431 cells, with β-actin as a loading control.

As shown above, both cell suspension and dense cultures promote S100A7 expression and lead to YAP inactivity. We hypothesized that YAP may function as a repressor of S100A7 induction in A431 cells. To test this hypothesis, we overexpressed YAP wild type (YAP-WT) and YAP Serine 127 to alanine mutant (YAP-S127A) in suspended and dense cells. YAP-S127A is known to mainly localize in nucleus [15]. As expected, YAP-WT and YAP-S127A repressed suspension- and dense-induced S100A7 expression, and YAP-S127A has a great effect (Figure 2A). Thus, these results suggest that nuclear YAP is responsible for inhibition of S100A7 induction in A431 cells. Next, to examine whether YAP-repressed S100A7 expression was regulated by the Hippo pathway in suspended and dense cells, we knocked down LATS1 in these cells using siRNAs because YAP phosphorylation via Lats1/2 kinases can result in cytoplasmic translocation and its subsequent inactivity [15]. The knockdown efficiency of two different specific LATS1 siRNAs and MST1 siRNAs were detected by Western blot and qPCR (Supplementary Figure S2A and S2B). We chose the better siRNA to continue the following experiments. Of note, depletion of LATS1 markedly reduced suspension- and dense-induced YAP phosphorylation and attenuated S100A7 expression in these cells (Figure 2B). A similar effect of knockdown of MST1 on the inhibition of S100A7 expression and YAP phosphorylation was also observed in suspended and dense cells (Figure 2C). These results indicate that the Hippo-YAP pathway controls suspension- and dense-induced S100A7 expression in A431 cells. On the other hand, in attached cells, silencing the expression of YAP alone was sufficient to induce S100A7 (Figure 3A) and consistent with a decrease of *CYR61*, which confirmed by qPCR (Figure 3B). Similarity, overexpression of LATS1 also led to an increase in S100A7 expression and YAP phosphorylation in attached A431 cells (Figure 3C). These results support that activation of the Hippo pathway promotes S100A7 expression in A431 cells.

Next, to investigate whether TEAD is YAP co-activator for S100A7 induction, we overexpressed YAP-WT and YAP-S94A in attached cells because YAP-S94A was defective in TEAD activation. Importantly, we found that S100A7 expression was marginally decreased by YAP-S94A compared with the control cells (Figure 3D), indicating that through interaction with TEAD, YAP inhibited S100A7 expression. More importantly, transiently depletion of TEAD1 alone was sufficient to induce S100A7 expression and also inhibited *CYR61* expression in cells (Figure 3E, 3F), whereas knockdown of TEAD2/3/4 had not have the same effects (Supplementary Figure S3). These data illustrate that YAP/TEAD1 but not TEAD2/3/4 is able to specifically repress S100A7 induction in A431 cells.

### S100A7 induction is mediated by actin cytoskeleton via the hippo pathway

**Figure 4:**
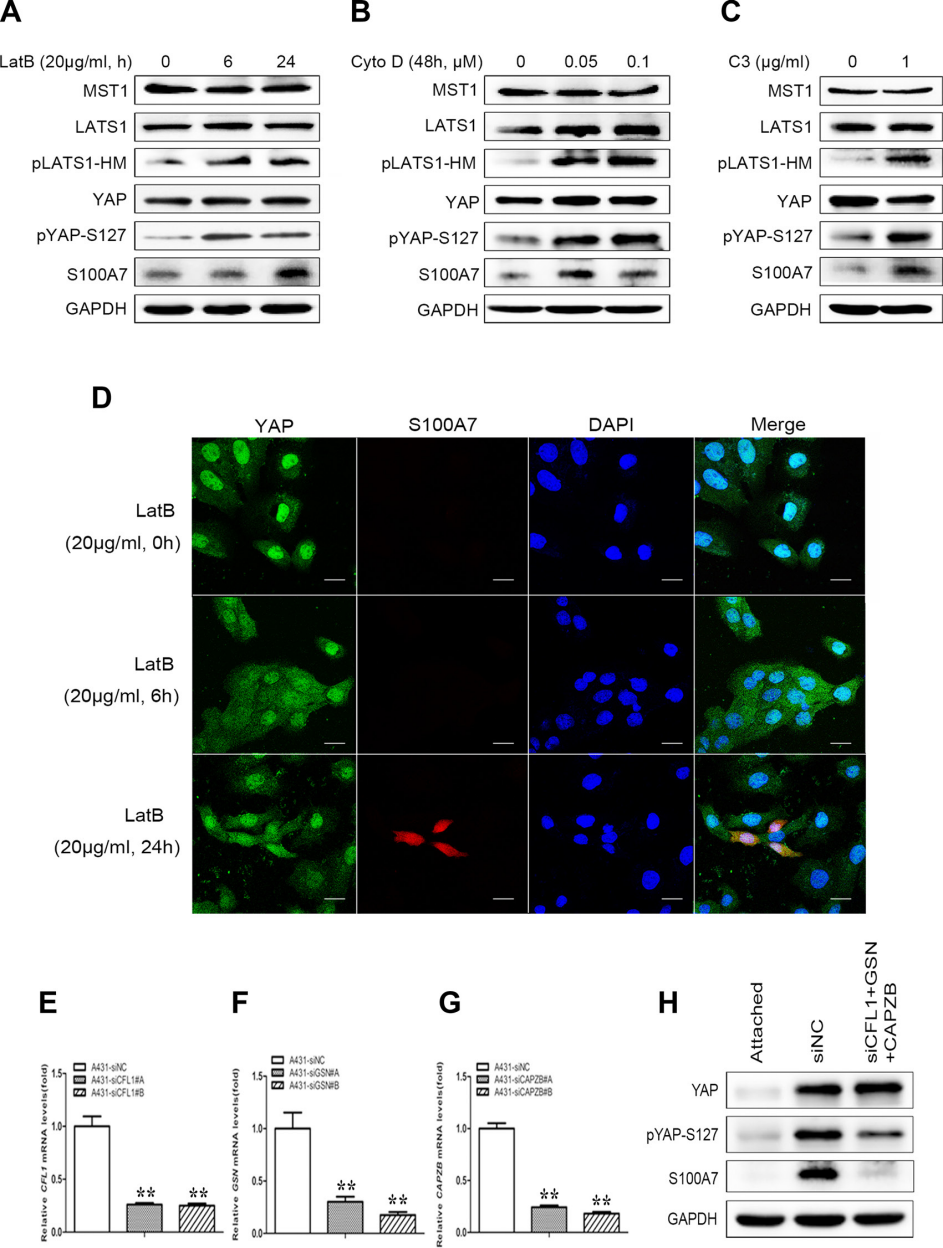
S100A7 induction is mediated by YAP via the actin cytoskeleton reorganization (**A**) A431 cells were treated with LatB (20 μg/ml) for 6 h or 24 h. (**B**) A431 cells were cultured for 48 h in the presence of Cyto D (0.05, 0.1 μM). (**C**) C3 (1 μg/ml) was added to the cells with serum-free growth medium for 4 h prior to harvesting for Western blotting analyses. (**D**) Subcellular location of S100A7 and YAP were detected in A431 cells treated with LatB. LatB induced S100A7 expression in a time-dependent manner and decreased nuclear YAP in A431 cells. Cells were treated with LatB (20 μg/ml) for different time. Samples were then stained with anti-S100A7 (Abcam) and anti-YAP (CST) antibodies. DAPI is a nuclear counterstain. Scale bar, 20 μm. Depletion of Cofilin1 (**E**), Gelsolin (**F**) and CAPZB (**G**) using siRNAs in normal attached A431 cells. The transfection efficiency of every two different specific siRNAs were detected by qPCR. Error bar, SD of three different experiments. *p < 0.05, **p < 0.01; *t*-test. (**H**) A431 cells were transfected with the indicated siRNAs simultaneously, and then were cultured under suspension condition for two days before the cells were harvested for Western blotting. GAPDH was used as a loading control.

Cell suspension and dense culture promoted YAP phosphorylation through actin cytoskeleton remodeling [20, 23]. If actin cytoskeleton reorganization also participates in YAP-mediated S100A7 induction in A431 cells, disruption of the actin cytoskeleton should also affect S100A7 expression. To confirm this hypothesis, two actin cytoskeleton-disrupting reagents, LatB and Cyto D were used to treat attached cells. As expected, abrogation of the actin polymerization by LatB and Cyto D resulted in S100A7 induction, similar to when cells are grown in suspension and dense conditions; this event also occurred in a LatB time-dependent and Cyto D dose-dependent manner. In agreement with findings obtained from previous reports [20, 21], an increase in LATS1 and YAP phosphorylation also occurred in A431 cells after treatment with LatB and Cyto D (Figure 4A and 4B). In addition, C3, a specific inhibitor of Rho, was also used for the reason that Rho also strongly induces YAP dephosphorylation via regulation of the actin cytoskeleton [20]. Indeed, C3 treatment significantly promoted the expression of S100A7 and phosphorylation of YAP and LATS1 (Figure 4C). On the other hand, by immunofluorescence, we observed that disruption of the actin cytoskeleton by Lat B decreased nuclear YAP and increased the percentage of S100A7-positive cells (Figure 4D). Together, the above results indicate that the actin cytoskeleton plays a critical role in mediating S100A7 expression via the Hippo pathway in A431 cells.

To further confirm whether F-actin plays an important role in S100A7 induction, we knocked down Cofilin1 (CFL1), Gelsolin (GSN) and CAPZB together in attached A431cells using their specific siRNAs (Figure 4E–4G) and then cultured cells in suspension for two days. As expected, S100A7 induction was significantly blocked and YAP-S127 was also markedly decreased in silenced-cells compared with control cells (Figure 4H). But if we silenced the three genes individually, the results were different (date not shown). Taken together, these data support that actin cytoskeleton reorganization plays an important role in S100A7 induction through activation of the Hippo pathway.

### Both S100A7 and YAP function as a promoting cell proliferation and inhibiting differentiation

**Figure 5:**
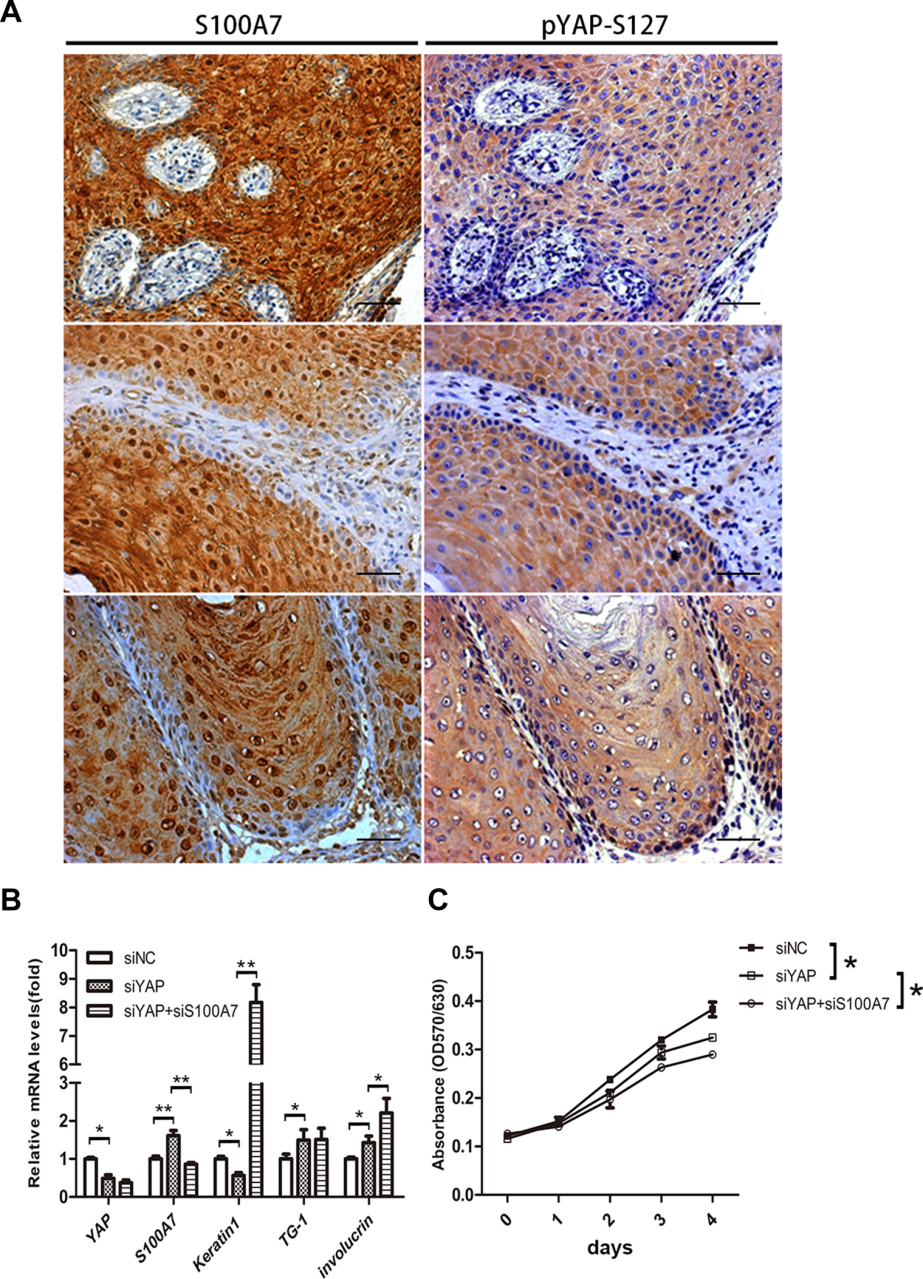
Identification of S100A7 and pYAP-S127 expression in skin SCC tissues, and loss of YAP and S100A7 leads to cell differentiation and growth inhibitions (**A**) Skin SCC tissues were examined by immunohistochemistry with specific anti-S100A7 and pYAP-S127 antibody. Scale bar, 50 μm. (**B**) Differentiation genes were induced by YAP, S100A7 deletion in A431 cells. mRNAs were isolated from A431 cells 48 h after infection with siRNAs, and were analyzed with real-time RT-PCR. Error bar, SD of three different experiments. *p < 0.05,**p < 0.01; *t*-test. (**C**) MTT assay showing depressed density of A431 cells after knockdown of YAP, or YAP and S100A7 simultaneously. Error bar, SD of three different experiments. *p < 0.05, **p < 0.01; *t*-test.

To test whether the negative correlation of S100A7 and YAP occurs in skin SCC tissues, we examined the expression pattern of S100A7 and pYAP-S127 in two consecutive sections of skin SCC tissue microarrays. Of the 72 skin SCC specimens we evaluated, 90.2% (65/72) were well or moderately differentiated carcinomas and the sixty-four specimens were positive for S100A7 expression, whereas none of the six poorly differentiated carcinomas expressed S100A7. In agreement with the results obtained *in vitro*, we found that cytoplasmic YAP-S127 staining was almost overlapped with the positive staining of S100A7 (Figure 5A). Importantly, the functional study showed that double deletion of S100A7 and YAP inhibited cell proliferation and promoted cell differentiation more effective than single deletion of YAP in A431 cells (Figure 5B and 5C). Based on our previous reports revealed that S100A7 promoted A431 cells proliferation and inhibited differentiation [12, 13], these results made a clue that both S100A7 and YAP may play the same or similar roles in A431 cells. Although S100A7 expression was inversely correlated with YAP activity in A431 cells and tissues, we guess that S100A7 may function as a substitute for YAP depending on cells microenvironment.

## DISCUSSION

Recently, cell morphology and cell density were found to regulate the Hippo pathway YAP through the actin cytoskeleton reorganization in human cells [24]. In the present study, we demonstrated for the first time that S100A7 is repressed by YAP via the Hippo pathway in A431 cells. In support of our conclusion, we identified that cell suspension and dense culture resulted in S100A7 induction and promoted phosphorylation of YAP (S127). Although an increase in YAP expression was also found in suspended and dense cells, the inhibition of YAP activity was confirmed by a decrease in *CYR61* expression. These results provided three important clues. First, the simultaneous regulation of S100A7 induction and YAP phosphorylation by suspension and dense culture suggested the involvement of the Hippo pathway and the actin cytoskeleton. Second, the dynamic expression of S100A7 was inversely correlated with YAP activity. Third, YAP may be a repressor of S100A7 expression in A431 cells. Later study indeed demonstrated that activation of the Hippo pathway by overexpression of LATS1 or knockdown of endogenous YAP significantly promoted S100A7 induction in A431 cells. In contrast, inactivation of the Hippo pathway by depletion of LATS1 and MST1 markedly blocked suspension- and dense-induced S100A7 expression and YAP phosphorylation. Similarly, S100A7 induction was partially inhibited by YAP-WT and better inhibited by YAP-S127A overexpression. Taken together, our findings reveal that the repressor function of YAP on S100A7 induction is regulated in a Hippo pathway-dependent manner in A431 cells: when the Hippo pathway is activated, the cells begin to induce S1007 expression, whereas inhibition of the Hippo pathway causes the cells to suppress S100A7 expression.

Next, we aim to investigate how nuclear YAP represses S100A7 expression in A431 cells. Although several transcription factors, including ErbB4, Runx2, and TEAD, have been reported to interact with YAP, TEAD represent the major target transcription factors of YAP [25–29]. As expected, we found that depletion of TEAD1 but not TEAD2/3/4 was sufficient to induce S100A7 expression and repressed *CYR61* expression in A431 cells. These results suggested that YAP-TEAD1 complex not only participated in gene activation but also gene repression. Similar to repression function of YAP/TEAD in this present study, a recent study has also provided direct evidence that YAP/TAZ-TEAD function as transcriptional co-repressors via recruitment of the nucleosome remodeling and histone deacetylase (NuRD) complex to target gene, and this repressor function requires TEAD1/3/4 transcription [22]. In addition, the YAP/TEAD has been proposed to mediate the repressor activity of Smad3 in human ES cells [30]. Conversely, YAP-TAED recruits the NcoA6 H3K4 methyltransferase complex and SWI/SNF chromatin-remodeling complex for target gene activation [31, 32]. Thus, our studies provide the first biological evidence that S100A7 induction is repressed by activation of the Hippo pathway via YAP/TEAD1 interaction in A431 cells, and our results significantly improve the understanding of the transcriptional co-repressor of YAP-TEAD in cells.

Cell suspension and dense could activate the Hippo pathway through actin cytoskeleton reorganization. If the Hippo-YAP was also involved in actin cytoskeleton reorganization-mediated S100A7 expression, depletion of F-actin depolymerizing and severing proteins should block actin cytoskeleton remodeling and restored YAP activity and S100A7 inhibition in suspended cells. As expected, when suspended cells were treated by triple knockdown of CAPZB, GSN and CFL1, YAP phosphorylation and S100A7 expression were markedly decreased compared with the control group. On the other hand, we treated the normal attached cells with LatB, Cyto D and C3 to disrupt the F-actin and ‘trick’ these cells to behave as if they grew in suspension or confluence. We found that both S100A7 expression and YAP phosphorylation were significantly increased in the attached cells after pharmacological perturbation of the actin cytoskeleton. Taken together, we provided compelling evidence that the integrity of the actin cytoskeleton plays a key role in regulation of S100A7 expression via YAP.

Besides the actin cytoskeleton, it has been also found that disruption of microtubule polymerization by nocodazole strongly blocked detachment-induced YAP phosphorylation but not affected attachment-induced YAP dephosphorylation [20]. In addition, the Hippo-YAP pathway is also regulated by G-protein coupled receptor signaling and protease activated receptor PAR [33]. Recently, several studies provide evidence that disruption of the E-cadherin-catenin complex at cell-cell junction also leads to activation of YAP [34]. Therefore, except of the actin cytoskeleton, S100A7 may also be regulated by other signaling pathways through the Hippo pathway. Although the expression of YAP and S100A7 displaying the negative correlation in A431 cells and skin SCC tissues, we have demonstrate that both play the similar function in cell proliferation and differentiation. Thus, we guess that S100A7 may function as a substitute for YAP in cells suspension and confluence culture in order to maintain cell survive or inhibit cell anoikis.

In summary, our findings prove a novel function of the Hippo-YAP pathway in regulation of S100A7 expression in A431 cells and provide a novel strategy for S100A7-regualted cancer therapy. The precise function of S100A7 and YAP in SCC cells (A431 cells) are going on in our laboratory.

## MATERIALS AND METHODS

### Cell culture

Human carcinoma cell line A431 was purchased from the Chinese Academy of Sciences Committee Type Culture Collection Cell Bank and was authenticated by short tandem repeat analysis at HK Gene Science Technology Co (Beijing, China). All cells were cultured according to the corresponding culture methods of the ATCC. Cell suspension cultures were obtained as described in our previous studies [12]. Cultures with different cell densities were achieved by plating cells at low cell density (here-after called ‘sparse’, 10000 cells/cm^2^) and at high cell density (‘dense’, 100 000 cells/cm^2^).

### Tissue specimens

72 cases of skin SCC tissues was obtained from one tissue microarray (No.SK802a) purchased from Xi'an Alenabio Company (Xian, China). All cancer patients had received a pathological diagnosis and none had received prior therapy. All cancer tissues were obtained from surgically treated patients who gave their informed consent. The study was approved by the Medical Ethics and Human Clinical Trial Committee at Henan Tongxu County People's Hospital.

### Plasmids and reagents

The pcDNA4-His-YAP WT; S127A and pCMV14-Flag-YAP WT; S94A vectors were kindly provided by Dr. Zhang (Mayo Clinic College of Medicine, USA). For pCMV14-Flag-LATS1, the LATS1 (NCBI Gene ID: 9113) cDNA fragment was amplified using 5′-CGGGGTACCATGAAGAGGAGTGAAAAG-3′and 5′-GCTCTAGAAACATATACTAGATCGCGATTT-3′, and then was cloned into the mammalian expression vector pCMV14 (Invitrogen, Carlsbad, CA, USA) using *KpnI* and *XbaI* restriction enzymes (Takara). Latrunculin B (L5288) and Cytochalasin D (C8273) were purchased from Sigma. Botulinum toxin C3 (CT04) was purchased from Cytoskeleton.

### siRNA and transfection

To silence the expression of YAP, LATS1, MST1, Cofilin1, Gelsolin, CAPZB and TEAD1, TEAD2, TEAD3 and TEAD4, all siRNAs as well as the non-targeting control siRNA were purchased from Gene Pharma and transfected using the Transfection Reagent (Polyplus) according to the manufacturer's protocol. For each gene, two individual siRNAs were used (Supplementary Table S1).

### Western blot

Western blotting analysis was performed as previously described [35]. The following antibodies were used: S100A7 (1/1000; Abcam, ab13680); YAP (1/500, Santa Cruz, sc-101199); pYAP (S127) (1/1000; Cell Signaling Technology, 13008S); anti-Flag tag (CWBIO, CW0287A); anti-His tag (MBL, D291-3). GAPDH (ZSGB-BIO, TA-08) and β-actin (ZSGB-BIO, TA-09) were used as loading controls.

### Reverse transcription and quantitative RT-PCR

Total RNA was extracted from cells for the generation of single-stranded cDNA. Quantitative RT-PCR (qPCR) was performed using an ABI 7300 Real-time PCR System (Applied Biosystems) with the Power SYBRÒ Green PCR Master Mix (Applied Biosystems) in a final volume of 20 μL. *GAPDH* was used as an endogenous control for each sample. The primers used for each of the genes are listed (Supplementary Table S2).

### Immunofluorescence staining

To examine the expression pattern of S100A7 and YAP in dense cells, cells were plated on coverslips at sparse or dense and then cultured for 6 and 24 h. Immunofluorescence staining was performed as described in our previous study [12]. The targeted proteins were detected using confocal microscopy (ZEISS LSM700, Germany) and a ZEISS LSM700 laser-scanning confocal microscope image system. Nonspecific IgG was used as a negative control.

### Immunohistochemistry

Immunohistochemistry was performed as described in our previous study [36]. Anti-S100A7 (1/200) and anti-pYAP-S127 (1/200) were separately incubated with the specimens. The goat anti-Rabbit/Mouse secondary antibody was purchased from MAIXINBIO (KIT-5010). S100A7 and pYAP-S127 expression was detected using a ZEISS ImagerA1 light microscope (Germany).

### MTT cell proliferation assay

Cells were trypsinized 24 h post-siRNA transfection, transferred to a 96-well plate in triplicate. Cell proliferation rate were evaluated by MTT.

### Statistical analysis

All of the experiments were repeated at least twice. Statistical analysis was performed using GraphPad Prism software. The statistical significance was evaluated using Student's *t*-test (2-tailed) to compare two groups of data. The asterisks indicate significant differences between the experimental groups and corresponding control condition. Differences were considered statistically significant at a *p*-value of less than 0.05. *p < 0.05, **p < 0.01.

## SUPPLEMENTARY MATERIALS FIGURES AND TABLES


