# Supplementary material for: S100A7 induction is repressed by YAP via the Hippo pathway in A431 cells
Source: Oncotarget. 2016 May 19;7(25):38133–42. doi: 10.18632/oncotarget.9477 (PMC5122377; doi:10.18632/oncotarget.9477)
Supplement: Supplementary file 1 [file oncotarget-07-38133-s001.pdf]

S100A7 induction is repressed by YAP via the Hippo pathway in A431 cells

Supplementary Materials

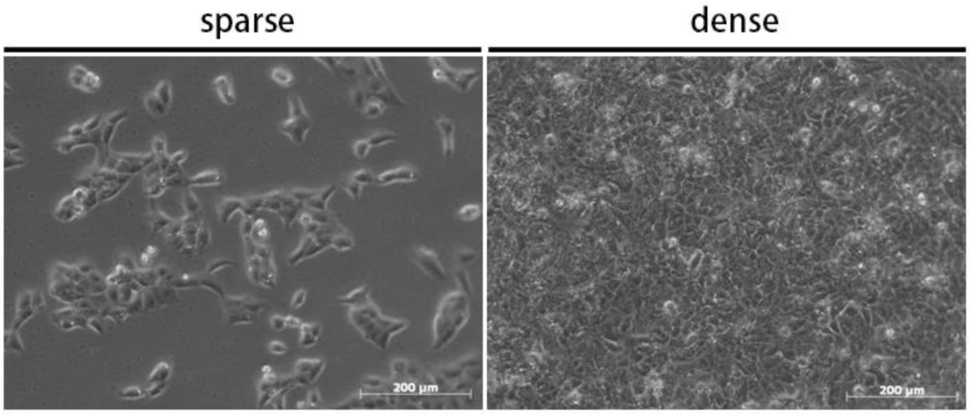

Supplementary Figure S1: Cell morphology of A431 cells in sparse and dense culture. A431 cells were seeded to obtain sparse and dense cells. Sparse: 10000 cells/cm^2; Dense: 100 000 cells/cm^2. After two days, cells were visualized by AxioObserverD1. Scale bar, 200 µm.

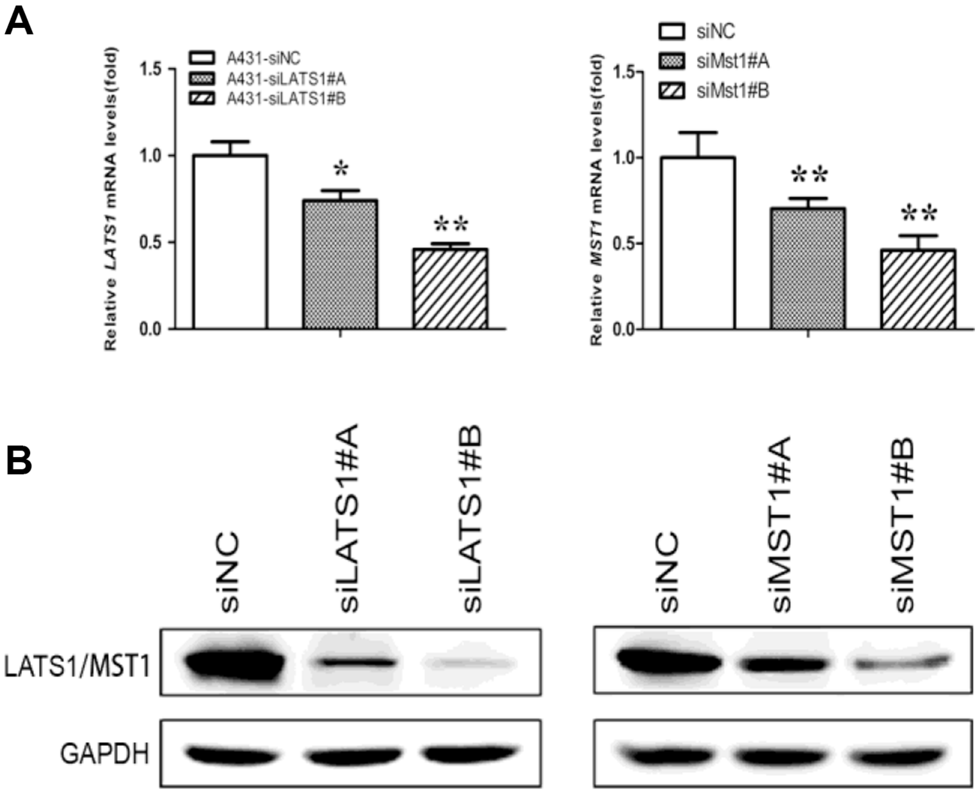

Supplementary Figure S2: Depletion of LATS1 and MST1 using siRNA in A431 cells. (**A**, **B**) The knockdown efficiency of two different specific siRNAs were detected by qPCR and Western blot. Error bar, SD of three different experiments. $*p < 0.05$, $**p < 0.01$; *t*-test.

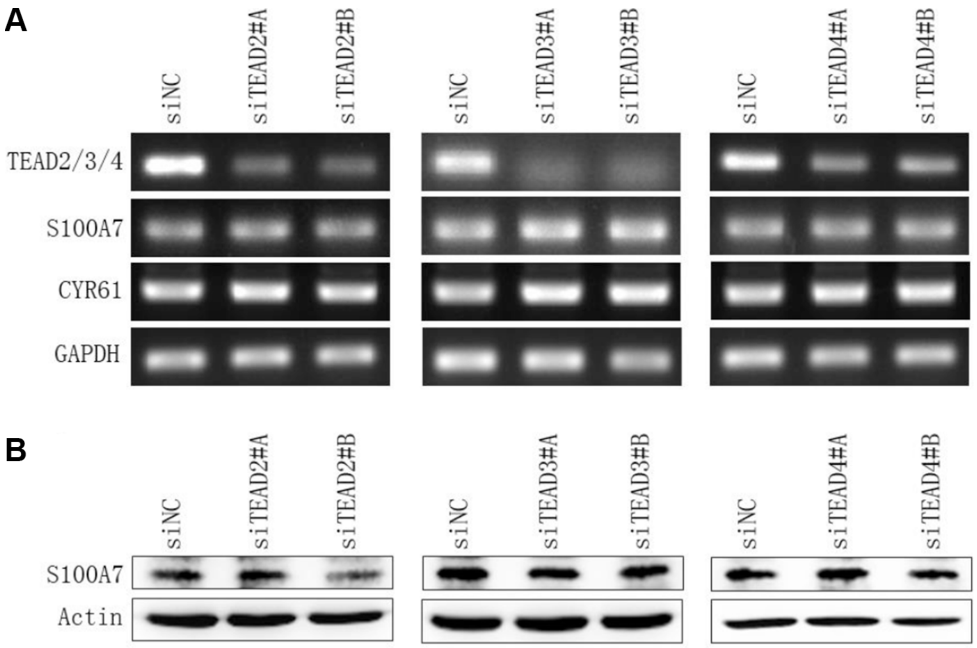

Supplementary Figure S3: Depletion of TEAD2/3/4 using siRNAs in A431 cells. The knockdown efficiency of two different specific siRNAs were detected by PCR (**A**), and the expression of S100A7 and CYR61 were detected by PCR and Western blot (**A** and **B**).

Supplementary Table S1: siRNA sequences

Genes name	siRNA sequences
YAP-sense	5′GGUGAUACUAUCAACCAAATT3′
YAP-antisense	5′UUUGGUUGAUAGUAUCACCTT3′
LATS1#A-sense	5′GAGCUGGAAAGGUU CUAAATT3′
LATS1#A-antisense	5′UUUAGAACCUUUCCAGCUCTT3′
LATS1#B-sense	5′GCAGCGU CUACAU CGUAAATT3′
LATS1#B-antisense	5′UUUACGAUGUAGACGCUGCTT3′
MST1#A-sense	5′GGACCUGCAUCAUGAACAATT3′
MST1#A-antisense	5′UUGUUCAUGAUGCAGGUCCTT3′
MST1#B-sense	5′GCUUCUCCUCCUGCCAUAUTT3′
MST1#B -antisense	5 AUAUGGCAGGAGGAGAAGCTT3′
CFL1#A -sense	5′CCACCUUUGUCAAGAUGCUTT3′
CFL1#A -antisense	5′AGCAUCUUGACAAAGGUGGTT3′
CFL1#B -sense	5′GGUGUCAUCAAGGU GUUCATT3′
CFL1#B -antisense	5′UGAACACCUUGAUGACACCTT3′
GSN#A-sense	5′GCGACAGCUACAU CAUU CUTT3′
GSN#A-antisense	5′AGAAUGAUGUAGCUGUCGCTT3′
GSN#B-sense	5′CUGGGUUGGAAAGGAUUCUTT3′
GSN#B-antisense	5′AGAAUCCUUUCCAACCCAGTT3′
CAPZB#A-sense	5′GCUGGAGUGAU CCU CAUAATT3′
CAPZB#A-antisense	5′UUAUGAGGAU CACU CCAGCTT3′
CAPZB#B-sense	5′GUACGCUGAACGAGAUCUATT3′
CAPZB#B-antisense	5′UAGAU CU CGUU CAGCGUACTT3′
TEAD 1#A-sense	5′GCCACUGCCAUUCAUAACATT3′
TEAD1#A-antisense	5′UGUUAUGAAUGGCAGUGGCTT3′
TEAD1#B-sense	5′AUGGCCGAUUUGUAUACCGAATT3′
TEAD1#B-antisense	5′UU CGGUAUACAAAU CGGCCAUTT3′
TEAD2#A-sense	5′GCCAGAUGCAGUUGAUUCUTT3′
TEAD2#A-antisense	5′AGAAUCAACUGCAUCUGGCTT3′
TEAD2#B -sense	5′CGGCAGAUCUACGACAAAUTT3′
TEAD2#B-antisense	5′AUUUGUCGUAGAUCUGCCGTT3′
TEAD3#A-sense	5′CCAGUGUCCUGAAGAACAATT3′
TEAD3#A-antisense	5′UUGUUCUGCAGGACACUGGTT3′
TEAD3#B-sense	5′GACCCU CU CAGGACAU CAATT3′
TEAD3#B-antisense	5′UUGAUGUCCUGAGAGGGUCTT3′
TEAD4#A-sense	5′CCGCCAAAUCUAUGACAAATT3′
TEAD4#A-antisense	5′UUUGUCAUAGAUUUGGCGGTT3′
TEAD4#B -sense	5′CCACGAAGGUCUGCUCUUUTT3′
TEAD4#B-antisense	5′AAAGAGCAGACCUUCGUGGTT3′

Supplementary Table S2: Primers used for qPCR

Genes name	Primers sequences
S100A7-sense	5′CTTCCCCAACTTCCTTAGTG 3′
S100A7-antisense	5′GTAGTCTGTGGCTATGTCTC 3′
CYR61-sense	5′GCTGCGAGGAGTGGGTCTGT 3′
CYR61-antisense	5′GGGTT GT AT AGGAT GCGAGGCT 3′
CTGF-sense	5 GCATCCGTACTCCCAAAATCTC 3′
CTGF-antisense	5′CAGGGCACTTGAACTCCACC 3′
GAPDH -sense	5′GAGT CAACGGATTTGGT CGT 3′
GAPDH-antisense	5′GACAAGCTTCCCGTTCTCAG 3′
YAP-sense	5′CCTCTATTTGCTCTTCCTTGTCC 3′
YAP-antisense	5′CCATCATCCAAACAGGCTCAC 3′
LATS1-sense	5′CACCCTTCTTGGATACCACAGC 3′
LATS1-antisense	5′CTGATTGACTCGTATGGAGGAACA 3′
MST1-sense	5′TGCTTCTGACTCAATGCTTAGGG 3′
MST1-antisense	5′TGGCTGCTCACGTTGTAGTGG 3′
CFL1-sense	5′GACTGCCGCTATGCCCTCTA 3′
CFL1-antisense	5′TGCAATTCATGCTTGATCCCT 3′
GSN-sense	5′TCCAACGATGCCTTTGTTCTGA 3′
GSN-antisense	5′ CATCTGGCTCGCTGCCTTCT 3′
CAPZB-sense	5′TGGACTGTGCCTTGGACCTAA 3′
CAPZB-antisense	5′TAATCCTTTCCCACCACCTTGT 3′
TEAD1-sense	5′TCGAGCAGCAGCGAGACCCAGACTC 3′
TEAD1 -antisense	5′TTACGAGGAAGAAGGCATTTTGAGG 3′
TEAD2-sense	5′TGCCTTCTTCCTGGTCAAGTTCTG 3′
TEAD2-antisense	5′CTCATACTGGCTGCTCACTCCGT 3′
TEAD3-sense	5′TCCTGTCAGACGAGGGCAAGATG 3′
TEAD3-antisense	5′CTTCCGAGCTAGAACCTGTATGTG 3′
TEAD4-sense	5′TTGAGCAGAGTTTCCAGGAGGCC 3′
TEAD4-antisense	5′CAATCAGCTCGTTCCGACCATACA 3′
